# Electroacupuncture Promotes Liver Regeneration by Activating DMV Acetylcholinergic Neurons‐Vagus‐Macrophage Axis in 70% Partial Hepatectomy of Mice

**DOI:** 10.1002/advs.202402856

**Published:** 2024-06-25

**Authors:** Liu Yang, Yanyu Zhou, Zhaoshuai Huang, Wenxuan Li, Jiacheng Lin, Weifan Huang, Yali Sang, Fang Wang, Xuehua Sun, Jiangang Song, Hailong Wu, Xiaoni Kong

**Affiliations:** ^1^ Central Laboratory Shuguang Hospital Affiliated to Shanghai University of Traditional Chinese Medicine Shanghai 201203 China; ^2^ Abdominal Transplantation Center General Surgery Ruijin Hospital School of Medicine Shanghai Jiao Tong University Shanghai 201203 China; ^3^ Department of anaesthesiology Shuguang Hospital Affiliated to Shanghai University of Traditional Chinese Medicine Shanghai 201203 China; ^4^ Shanghai Key Laboratory of Molecular Imaging Collaborative Innovation Center for Biomedicines Shanghai University of Medicine and Health Sciences Shanghai 201203 China

**Keywords:** acetylcholinergic neurons, dorsal motor nucleus of vagus, electroacupuncture, liver regeneration, vagus nerve

## Abstract

Lack of liver regenerative capacity is the primary cause of hepatic failure and even mortality in patients undergoing hepatectomy, with no effective intervention strategies currently available. Therefore, identifying efficacious interventions to enhance liver regeneration is pivotal for optimizing clinical outcomes. Recent studies have demonstrated that vagotomy exerts an inhibitory effect on liver regeneration following partial hepatectomy, thereby substantiating the pivotal role played by the vagus nerve in the process of liver regeneration. In recent years, electroacupuncture (EA) has emerged as a non‐invasive technique for stimulating the vagus nerve. However, EA on hepatic regeneration remains uncertain. In this study, a 70% partial hepatectomy (PH) mouse model is utilized to investigate the effects of EA on acute liver regeneration and elucidate its underlying molecular mechanisms. It is observed that EA at ST36 acutely activated cholinergic neurons in the dorsal motor nucleus of the vagus nerve (DMV), resulting in increased release of acetylcholine from hepatic vagal nerve endings and subsequent activation of IL‐6 signaling in liver macrophages. Ultimately, these events promoted hepatocyte proliferation and facilitated liver regeneration. These findings provide insights into the fundamental brain‐liver axis mechanism through which EA promotes liver regeneration, offering a novel therapeutic approach for post‐hepatectomy liver regeneration disorders.

## Graphical abstract

Electroacupuncture (EA) at ST36 activates cholinergic neurons in the dorsal motor nucleus of the vagus nerve (DMV) of the brain, leading to the release of acetylcholine from hepatic vagus nerve terminals which subsequently activates IL‐6 signaling in liver macrophages and promotes liver regeneration.

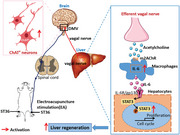

## 1 Introduction

The liver exhibits an extraordinary regenerative capacity, and in response to hepatic tissue loss caused by toxins or partial surgical resection, the remaining hepatocytes undergo substantial proliferation to restore the original weight and functionality of the liver.^[^ ^1^ ^]^ Given that China still faces a high prevalence of liver disease with a significant number of patients, liver resection and transplantation continue to be the primary treatment modalities for end‐stage liver disease.^[^ ^2^ ^]^ Despite the robust regenerative capacity of the liver, there exists a considerable proportion of patients with poor prognosis due to impaired regenerative ability, leading to liver failure and even mortality. For instance, in cases where acute (e.g., acetaminophen) or chronic insults (e.g., hepatitis B virus, hepatitis C virus, or non‐alcoholic fatty liver disease) damage the liver, its regenerative potential is greatly diminished.^[^ ^3^ ^]^ Therefore, identifying an effective intervention strategy to promote liver regeneration is crucial for improving clinical outcomes.

Liver regeneration represents a complex and well‐orchestrated physiological and pathological process within the context of multi‐system interactions in the human body.^[^ ^4^ ^]^ Previous studies have primarily focused on investigating factors such as inflammatory mediators, growth factors, metabolism regulation, and immune responses in relation to hepatic regeneration.^[^ ^5^ ^]^ However, little is known about the involvement of extrinsic factors beyond hepatic origin‐particularly those related to nervous system‐in modulating liver regeneration. Recently published research has provided an overview regarding our current understanding of hepatic nervous system development along with its implications in both liver regeneration and diseases. The innervation of the liver involves autonomic fibers as well as sensory fibers from sympathetic and parasympathetic divisions which intricately regulate various aspects including hepatic function maintenance, promotion of regenerative processes within the organ itself during injury or disease states.^[^ ^6^ ^]^ The literature suggests that branch vagotomy exerts an inhibitory effect on liver regeneration following PH, particularly during the early phases of the regenerative process.^[^ ^7^ ^]^ The evidence presented here demonstrates the pivotal involvement of the nervous system, particularly the vagus nerve, in the intricate process of liver regeneration.

Acupuncture and electroacupuncture (EA) are the integral component of traditional Chinese medicine, and its therapeutic efficacy has been clinically validated for millennia. As per the Traditional Medicine Strategy: 2014–2023 by the World Health Organization (WHO), acupuncture finds extensive utilization across 183 countries and territories, with numerous high‐quality clinical studies substantiating its effectiveness in diverse applications.^[^ ^8^ ^]^ The application of EA at the ST36 acupoint on the hind limb has been reported to stimulate the vagus‐adrenal axis in mice, thereby exerting an anti‐inflammatory effect.^[^ ^9^ ^]^ In recent years, the application of EA has emerged as a non‐invasive technique for vagus nerve stimulation.^[^ ^9^, ^10^ ^]^ Moreover, the vagus nerve activity is believed to potentially serve as a mediator in acupuncture treatment.^[^ ^11^ ^]^ However, the impact of EA on hepatic regeneration remains uncertain. Here, we employed 70% partial hepatectomy (PH) of mice as an experimental model to investigate the impact of EA on acute liver regeneration and elucidate its potential underlying molecular mechanisms. We found that EA at ST36 acutely activated cholinergic neurons in the dorsal motor nucleus of the vagus nerve (DMV), leading to increased release of acetylcholine from hepatic vagus nerve endings and subsequent activation of IL‐6 signaling in liver macrophages, ultimately promoting hepatocyte proliferation and liver regeneration. It elucidated the fundamental mechanism of the brain‐liver axis in the role of EA in promoting liver regeneration, thereby offering a novel therapeutic approach for post‐hepatectomy liver regeneration disorders.

## 2 Results

### 2.1 EA at the ST36 Acupoint Significantly Promoted Liver Regeneration after 70% Partial Hepatectomy of Mice

**Figure 1:**
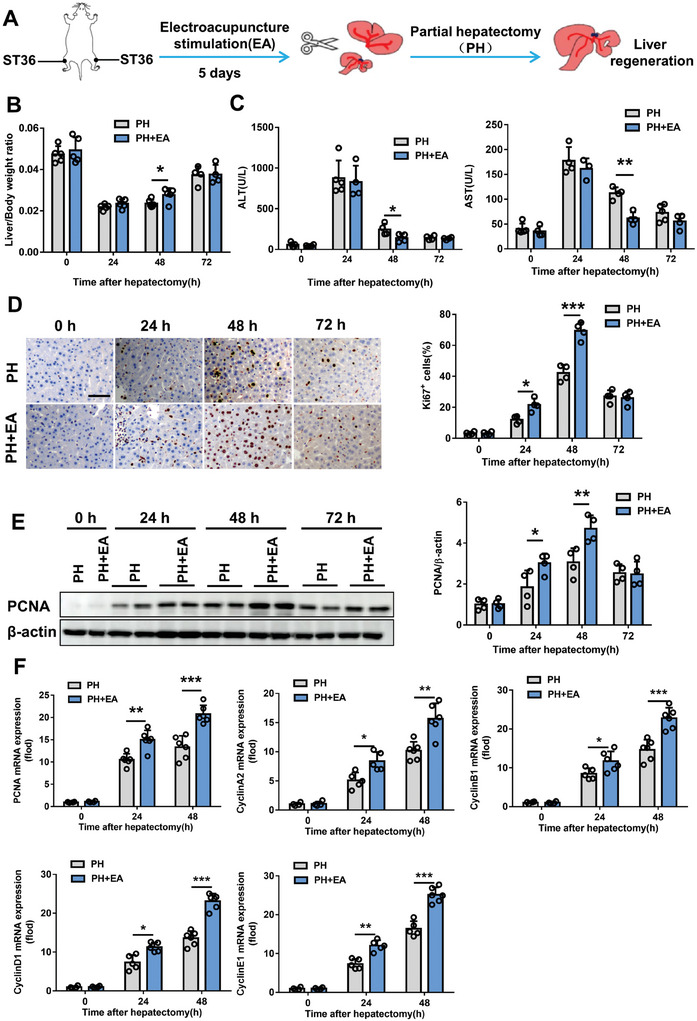
The effect of EA on liver regeneration was investigated in mice following 70% partial hepatectomy. A) A schematic diagram illustrating the procedure for electroacupuncture and 70% partial hepatectomy in mice. B) Liver/body weight ratio was measured at 0, 24, 48, and 72 h after the surgery to compare PH and EA+PH groups. C) Levels of alanine aminotransferase (ALT) and aspartate aminotransferase (AST) were assessed at different time points after the surgery to evaluate liver function in both groups. D) Immunohistochemical staining for Ki67 was performed to examine cell proliferation in the livers of PH and EA+PH mice at various time points post‐surgery. Scale bars, 20 µm. E) Western blot analysis was conducted to measure PCNA protein expression levels in liver tissue at different time points after the surgery. F) mRNA expression levels of PCNA, CyclinA2, CyclinB1, CyclinD1, and CyclinE1 genes were determined by qpcr in the livers at various time points following 70% PH. *n* = 5. Data are expressed as mean ± SD. *, *P* < 0.05; **, *P* < 0.01***; *P* < 0.001. The experiments above were repeated three times.

EA was administered at a frequency of 2/100 Hz and an intensity of 0.5 mA for 15 min per day over a period of 5 days. We employed the model of 70% PH to investigate the impact of EA on liver regeneration specifically targeting the hindlimb ST36 acupoint (**Figure** **1**A). The liver‐to‐body weight ratio was higher in the EA group compared to the PH group, with noticeable differences observed at 48 h post‐PH (Figure 1B). Moreover, the EA group exhibited improved liver function as evidenced by lower levels of alanine aminotransferase (ALT) and aspartate aminotransferase (AST) at 48 h after PH (Figure 1C). Consistently, Ki67‐positive hepatocytes were significantly increased in EA mice at both time points 24 and 48 h following PH when compared to sham‐operated mice (Figure 1D). Subsequently, similar outcomes were obtained through protein expression analysis of proliferating cell nuclear antigen (PCNA) (Figure 1E). Notably, PCNA along with Cyclin A2, Cyclin B1, Cyclin D1, and Cyclin E1 gene expressions were significantly upregulated in the livers from EA‐treated animals compared to those from sham‐operated animals at both time points 24 and 48 h after PH (Figure 1F). These findings collectively demonstrate that EA exerts significant effects in promoting liver regeneration subsequent to undergoing a procedure involving removal of 70% hepatic tissue.

### 2.2 EA Promoted Liver Regeneration by Activating Acetylcholinergic Neurons in DMV of Brain

**Figure 2:**
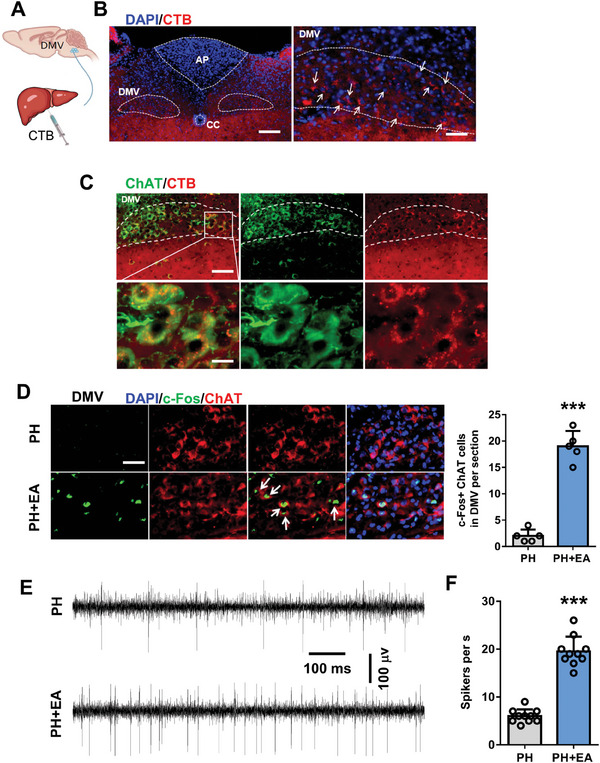
EA activated acetylcholinergic (Ach) neurons in DMV region of brain. A) Schematic with CTB injection. B) Fluorescence projection of CTB in DMV region of brain. Scale bars, 100 µm; Scale bars, 40 µm. C) Representative image of fluorescence colocalization of CTB signal with ChAT in the DMV region of the brain. Scale bars, 50 µm; Scale bars, 10 µm. D) Representative images of fluorescence colocalization of c‐Fos and ChAT in DMV, and the number of c‐Fos positive cells. Scale bars, 20 µm. E,F) Electrophysiological recordings of the spike number of DMV neurons. *n* = 5. Data are expressed as mean ± SD. ***, *P* < 0.001. The experiments above were repeated three times.

**Figure 3:**
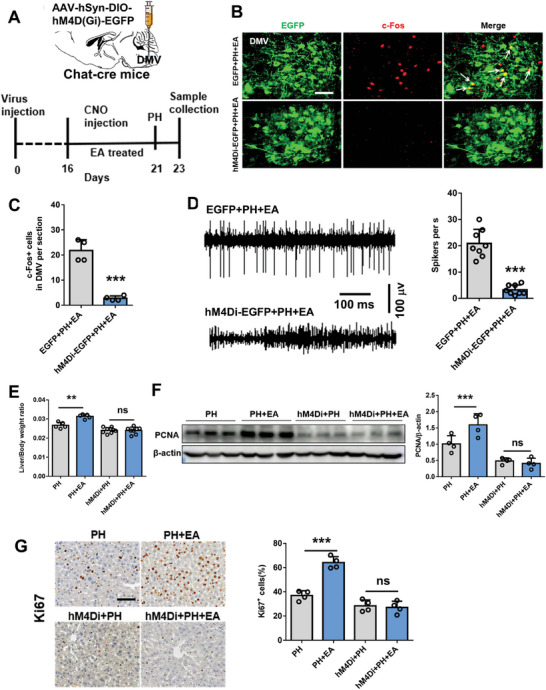
The effect of EA on liver regeneration is abolished after chemogenetic inhibition of Ach neurons in the DMV region. A) Schematic of chemogenetics and timeline of chemogenetic experiments. B,C) Representative DMV immunostaining of c‐Fos antibody in EGFP+PH+EA and hM4Di‐EGFP+ PH+EA mice 48 h after 70% PH and the quantification of c‐Fos+ neurons. Scale bars, 20 µm. D) Sample traces and action potential data of DMV neurons. E) liver/body weight ratio of PH, PH+EA, hM4Di+PH, and hM4Di+PH +EA groups 48 h after 70% PH. F) PCNA protein expression in liver tissue 48 h after 70% PH in four groups and the relative protein expression analysis. G) IHC staining of Ki67 48 h after 70% PH in four groups and the quantification of Ki67 positive cells. Scale bars, 20 µM. Data represent the mean ± SD. ns, *P* > 0.05; **, *P* < 0.01***; *P* < 0.001. The experiments above were repeated three times.

To investigate the potential liver‐brain connection, we injected cholera toxin subunit B (CTB) into the liver and traced it back to the brain a week later, observing a fluorescent signal in the cholinergic neuron‐rich DMV area,^[^ ^12^ ^]^ we observed colocalization of fluorescent signals with an anti‐choline acetyltransferase (ChAT) antibody (**Figure** **2**A–C). The results indicated that the acetylcholine neurons in the dorsal motor nucleus of the vagus nerve region had a direct regulatory effect on the hepatic nerves. Furthermore, EA increased c‐Fos expression in DMV and coincided with ChAT fluorescence signals (Figure 2D). Electrophysiological recordings showed a significant increase in spike number of DMV neurons in mice under EA compared to sham mice (Figure 2E,F). To verify the effect of Ach neurons on liver regeneration, we used chemogenetic inhibition to suppress neuronal firing using AAV‐hSyn‐DIO‐hM4D(Gi)‐EGFP virus and control virus (AAV‐DIO‐EGFP) injected into the DMV region (**Figure** **3**A). Inhibition was verified by c‐Fos immunofluorescence and electrophysiological recordings after 70% partial hepatectomy (Figure 3B–D). The liver/body weight ratio and protein expression levels of Ki67 and PCNA were similar between hM4Di+PH+EA and hM4Di+PH groups 48 h after PH, suggesting that EA promotes liver regeneration by activating acetylcholinergic neurons in the DMV region of brain (Figure 3E–G).

### 2.3 EA Exerts a Pro‐Hepatic Regenerative Effect Directly through Vagus‐Liver Rather Than Other Pathways

**Figure 4:**
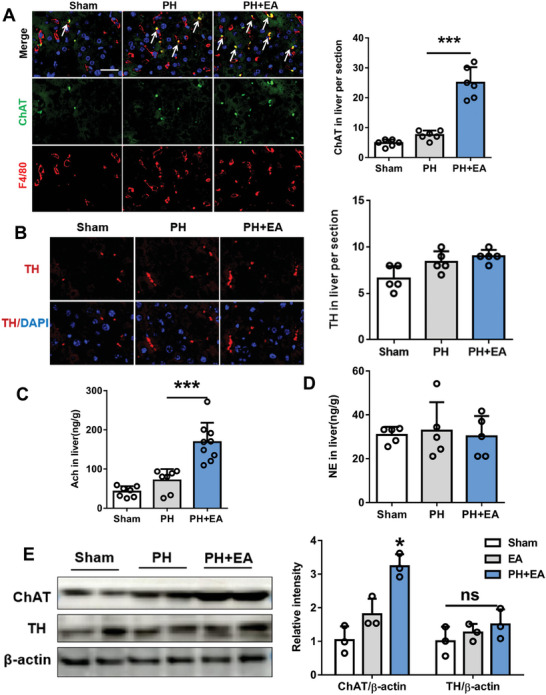
EA significantly activated the vagus nerve of liver, however had no effect on the sympathetic nerve. Immunostaining images and statistics of ChAT, F4/80 A) and TH B) in liver of sham, PH and PH+EA groups after 70% PH. Scale bars, 20 µm. Mass spectrometry detection of Ach C) and NE D) in liver of sham, PH and PH+EA groups after 70% PH. E)Western blot images and statistics of ChAT and TH in liver of sham, PH and PH+EA groups after 70% PH. Data represent the mean ± SD. ns, *P* > 0.05; ***; *P* < 0.001. The experiments above were repeated three times.

**Figure 5:**
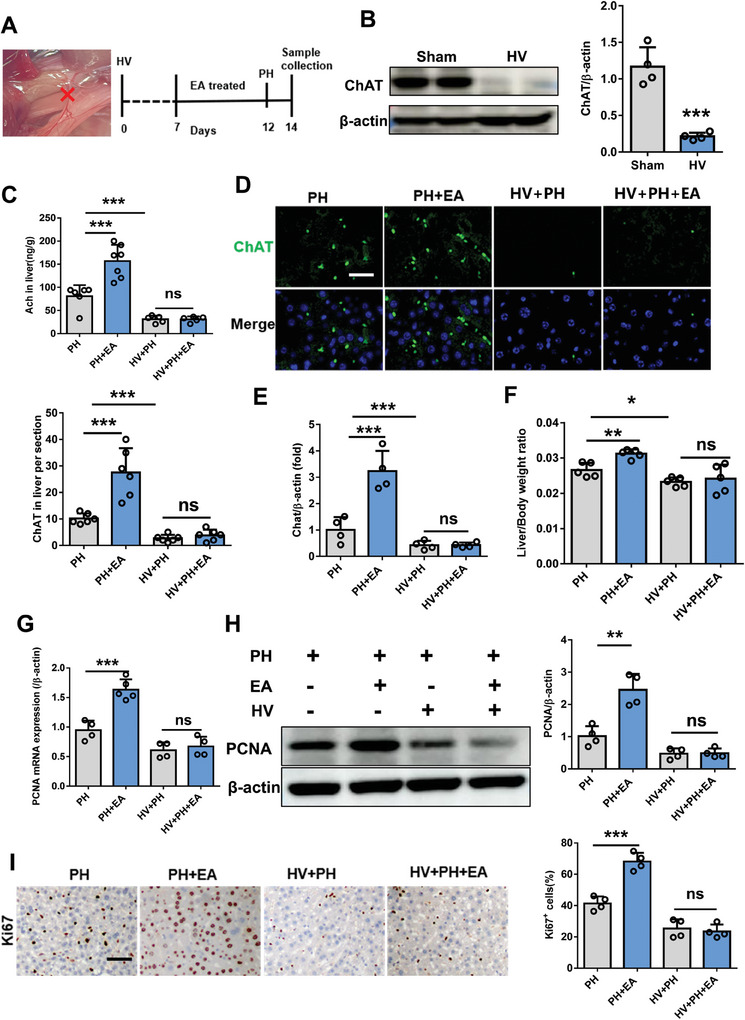
Hepatic branch vagotomy (HV) of mice eliminated the role of EA in promoting liver regeneration after 70% PH. A) Schematic of Hepatic branch HV in mice. B) The protein expression of ChAT and β−actinin in liver tissue were detected by western blot in sham and HV groups mice. C) Mass spectrometry detection of Ach in liver tissue of PH, PH+EA, HV+PH, HV+PH+EA groups after 70% PH. D) Representative immunostaining of ChAT antibody in PH, PH+EA, HV+PH, HV+PH+EA mice after 70% PH. Scale bars, 20 µm. E) The mRNA expression of chat at 48 h after 70% PH in mice from four PH, PH+EA, HV+PH, HV+PH+EA groups. F) liver/body weight ratio of mice in PH, PH+EA, HV+PH, HV+PH+EA groups after 48 h 70% PH. G) The mRNA expression of pcna at 48 h after 70% PH in mice from four PH, PH+EA, HV+PH, HV+PH+EA groups. H) PCNA protein expression in liver tissue 48 h after 70% PH in four groups and the relative protein expression analysis. I) IHC staining of Ki67 48 h after 70% PH in four groups and the quantification of Ki67 positive cells. Scale bars, 20 µm. Data represent the mean ± SD. ns, *P* > 0.05; *, *P* < 0.05; **, *P* < 0.01***; *P* < 0.001. The experiments above were repeated three times.

**Figure 6:**
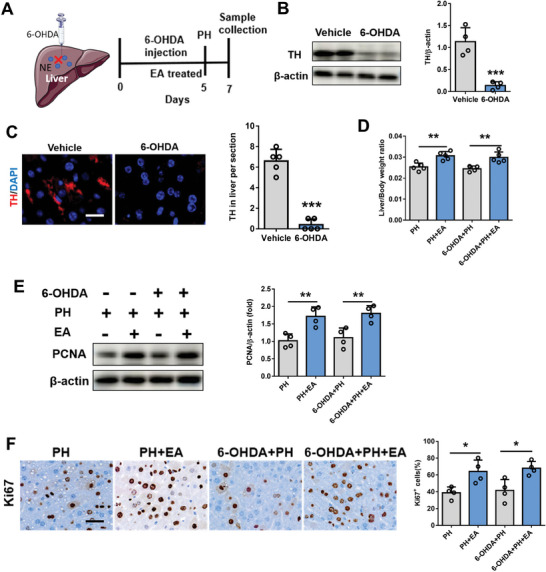
After the sympathetic nerve was removed, EA did not affect the promotion of liver regeneration after 70% PH. A) Schematic of 6‐OHDA administration in mice. B) Western blot test of TH in liver tissue in vehicle and 6‐OHDA administration mice. C) Representative immunostaining of TH antibody in vehicle and 6‐OHDA administration mice. Scale bars, 20 µm. D) liver/body weight ratio of mice in PH, PH+EA, 6‐OHDA+PH, 6‐OHDA+PH+EA groups after 48 h 70% PH. E) PCNA protein expression in liver tissue at 48 h after 70% PH in four groups by western blot and the relative protein expression analysis. F) IHC staining of Ki67 at 48 h after 70% PH in four groups and the quantification of Ki67 positive cells. Scale bars, 20 µm. Data represent the mean ± SD. *, *P* < 0.05; **, *P* < 0.01. The experiments above were repeated three times.

Considering that the liver projects acetylcholine neurons to the brain, we investigated the expression of acetylcholine neurons in the liver. Our findings revealed comparable levels of liver acetylcholine content between the sham and PH groups, as determined by immunofluorescence and mass spectrometry measurements. As shown in **Figure** **4**A, ChAT positive fibers within liver parenchyma and in close contact with liver macrophages (F4/80 positive cells). However, there was a significant increase in liver acetylcholine content in the PH+EA group (Figure 4A,C). Conversely, immunofluorescence and mass spectrometry results demonstrated no difference in norepinephrine (sympathetic secretion) expression within the liver among sham, PH, and PH+EA groups (Figure 4B,D). Consistent with these observations, western blot analysis also confirmed higher ChAT protein expression in the PH+EA group compared to both PH and sham groups; however, TH protein expression showed no significant change (Figure 4E). To further investigate the role of vagus nerve signaling, we assessed electroacupuncture's impact on liver regeneration following hepatic branch vagotomy (HV) in mice. Western blotting analyses along with mass spectrometry measurements, immunofluorescence staining assays and qPCR results collectively indicated a significant decrease in ChAT expression as an acetylcholinergic neuronal marker after HV (**Figure** **5**B–E). In line with previous findings, we observed a higher liver/body weight ratio in the PH+EA group compared to the PH group; however, no difference was noted between HV+PH+EA group versus HV+PH group (Figure 5F). Furthermore, evaluation of proliferation‐related markers PCNA and Ki67 yielded similar outcomes significantly increased protein expressions were observed for PCNA and Ki67 within the PH+EA group relative to those within only the PH group; whereas their expressions remained similar between HV+PH+EA and HV+PH groups (Figure 5G–I). To further verify the function of the sympathetic nerve, mice were administered the neurotoxin 6‐hydroxydopamine (6‐OHDA) to induce chemical sympathetic denervation (**Figure** **6**A). Administration of 6‐OHDA almost completely depleted the sympathetic nerve terminals in liver as revealed by immunofluorescence staining and western blot detection (Figure 6B,C). Further, mice were subjected to 70% PH model and we found that liver/body weight ratio 48 h after PH in 6‐OHDA mice without PH+EA was higher than that of PH mice (Figure 6D). Western blot and immunofluorescence staining results showed that the expression of PCNA and Ki67 protein in 6‐OHDA+PH+EA group was significantly higher than that in 6‐OHDA+PH group (Figure 6E,F). These results above suggested that EA promoted liver regeneration after 70% PH by activating the vagus nerve rather than the sympathetic nerve.

EA has been reported to exert systemic anti‐inflammatory effects via the vagus‐adrenal axis.^[^ ^9b^ ^]^ Therefore, we aimed to investigate whether EA exerts a direct regulatory effect on liver regeneration through the vagus nerve or an indirect effect via the vagal‐adrenal axis. First, we quantified serum corticosterone levels in sham‐operated, PH, and PH+EA groups using mass spectrometry analysis. The results revealed no significant differences in corticosterone levels among all experimental groups (Figure S1A, Supporting Information). Subsequently, we evaluated the impact of EA on liver regeneration following bilateral adrenalectomy (ADX) in mice. Mass spectrometry analysis demonstrated a marked reduction in serum corticosterone levels in adrenalectomized mice (Figure S1C, Supporting Information). Importantly, when considering the liver‐to‐body weight ratio and ki67 protein expression, the ADX+PH+EA group exhibited significantly higher values compared to the ADX+PH group; meanwhile, comparable results were observed between the PH+EA and ADX+PH+EA groups (Figure S1D–F, Supporting Information). In other words, adrenalectomy did not substantially affect the ability of EA to promote liver regeneration after 70% PH.

### 2.4 EA Promotes IL‐6 Release from Macrophages in the Early Stage of Liver Regeneration through Vagus Nerve

**Figure 7:**
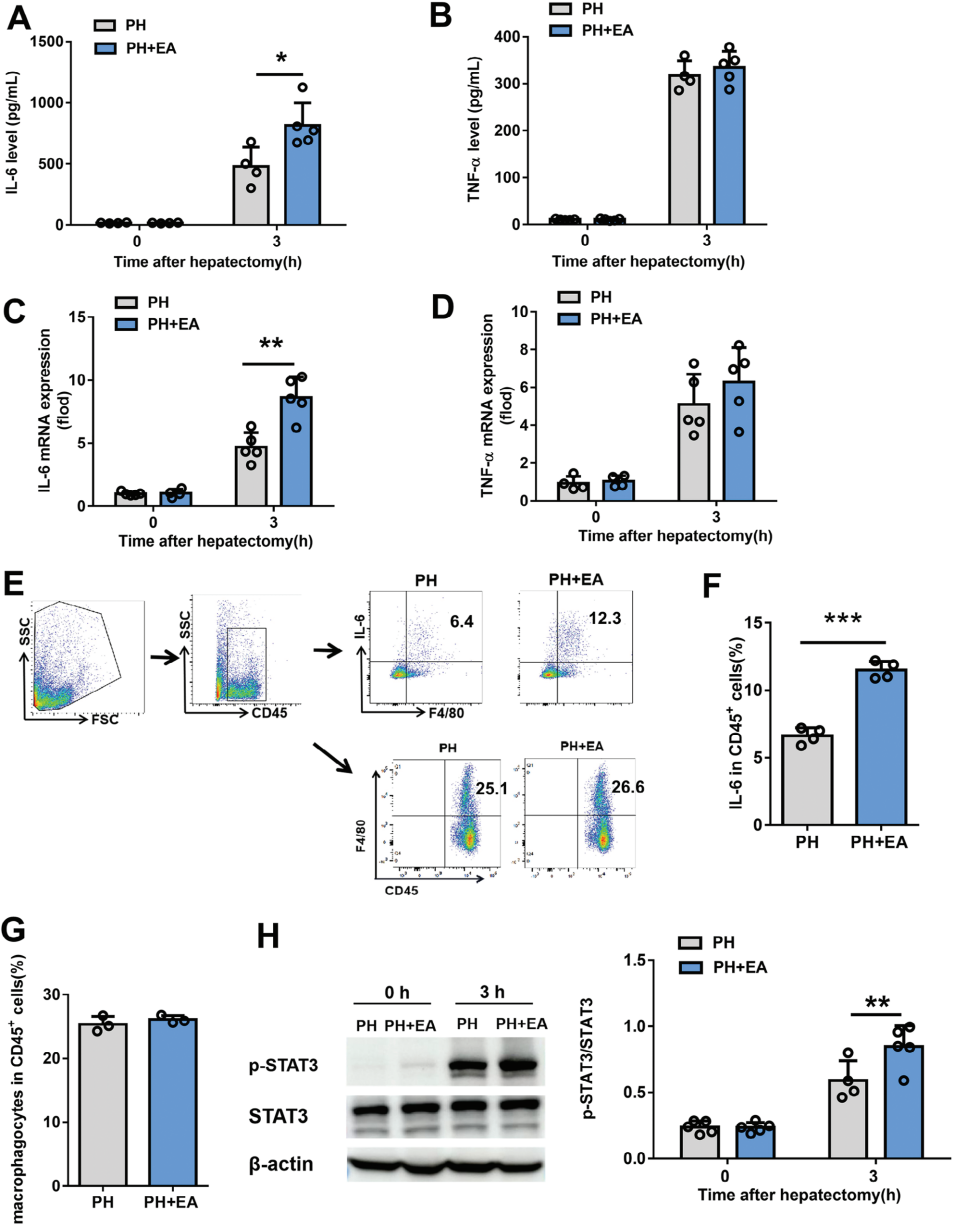
EA promotes IL‐6 release from macrophages in early liver regeneration after 70% PH. A,B) The secretion of IL‐6 and TNF‐α were detected by elisa in the remnant liver at 3 h after 70% PH. C,D) The mRNA expression of IL‐6 and TNF‐α genes in the liver tissue at 0, and 3 h after 70% PH. E) Representative FACS plots of hepatic leukocyte populations analyzed by flow cytometry analysis. The following gating strategies were used to identify various immune cells: hepatic macrophages (CD45^+^F4/80^+^), IL‐6 secretion of hepatic macrophages (CD45^+^F4/80^+^IL‐6^+^). Representative FACS plots of IL‐6^+^ hepatic macrophages and hepatic macrophages from PH and PH+EA mice at 3 h after PH and F,G) quantification of the proportion of these immune cell subtypes. H) Representative images of liver extract immunoblottings with anti‐phospho‐STAT3, total STAT3, and actin. Relative intensities of phospho/total STAT3 in livers from sham operation for PH and PH+EA mice. Data represent the mean ± SD. *, *P* < 0.05; **, *P* < 0.01; ***, *P* < 0.001. The experiments above were repeated three times.

**Figure 8:**
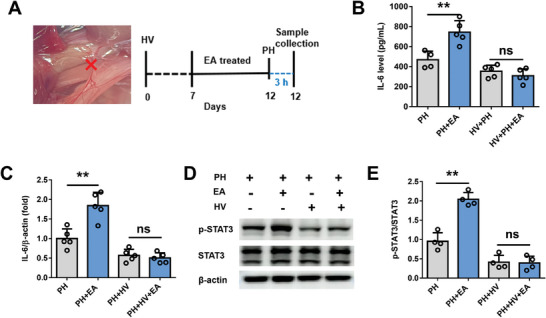
The enhancement effect of EA on IL‐6 disappeared after HV. A) Schematic of hepatic branch vagotomy in mice. B) The secretion of IL‐6 was detected by elisa from PH, PH+EA, HV+PH, HV+PH+EA mice in the remnant liver at 3 h after 70% PH. C) The mRNA expression of IL‐6 at 3 h after 70% PH in mice from PH, PH+EA, HV+PH, HV+PH+EA groups. D,E) Western blot assay for phospho‐STAT3, total STAT3, and β−actin protein expression in liver tissue at 48 h after 70% PH in four groups and the relative protein expression analysis.

So, what is the mediator of EA activation of the vagus‐liver link? It has been reported that the vagus nerve can increase IL‐6 production of resident macrophages in the liver.^[^ ^13^ ^]^ Then, we examined the effect of EA on IL‐6 secretion in the early stage of liver regeneration after 70% PH. We found that a serum IL‐6 content in the PH+EA group was significantly higher than that in the PH group at 3 h after 70% PH, while TNFα content was similar between the two groups by the elisa test (**Figure** **7**A,B). More importantly, both flow cytometry and qpcr detection indicated that more IL‐6 was released n in the remnant liver of EA‐treated mice at 3 h after 70% PH (Figure 7C,E,F). It is worth mentioning that the enhancement effect of EA on IL‐6 disappeared after HV (**Figure** **8**B,C). However, EA had little effect on TNF‐α secretion in the remnant liver at 3 h after 70% PH (Figure 7D). As we all known, IL‐6 exert its effects through signal transducer and activator of transcription 3 (STAT3) signaling. Next, liver STAT3 phosphorylation was examined in PH and PH+EA mice by western blot at 3 h after 70% PH, which was significantly increased in PH+EA mice compared with PH mice (Figure 7H), and this enhancement of phosphorylation was blunted by HV (Figure 8D,E). In addition, EA did not change the number of macrophages in the early stage of liver regeneration after 70% PH (Figure 7G), and there were no significant differences in percentages of neutrophils, T cells and B cells (Figure S2A–D, Supporting Information).

### 2.5 EA Promotes Liver Regeneration Depending on the Acetylcholine Signal Mediated by Liver Macrophages

**Figure 9:**
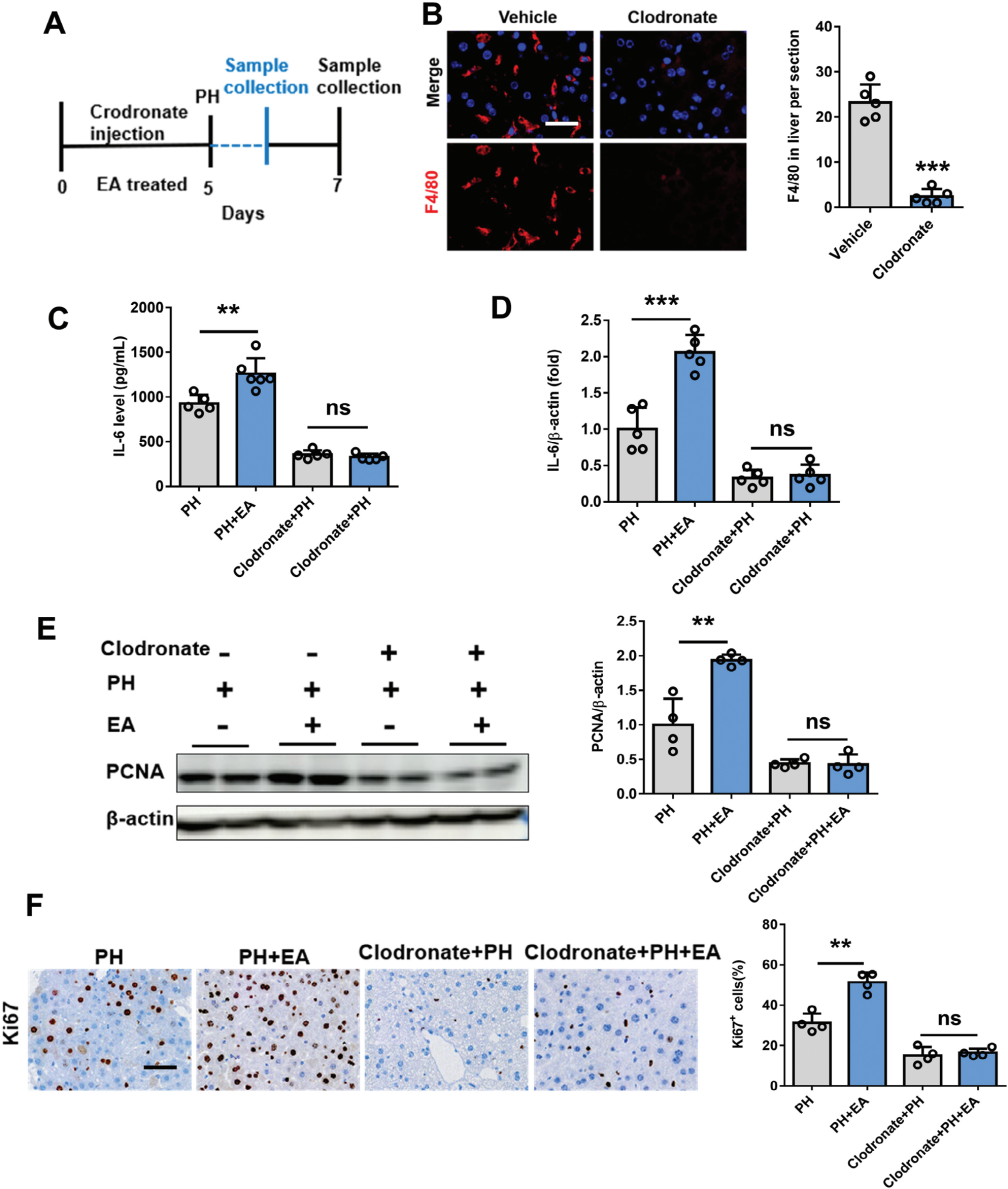
Clodronate administration eliminated the effect of EA on increasing IL‐6 secretion in the early stage of liver regeneration after 70% PH in mice. A) Schematic of crodronate injection in mice. B) Representative immunostaining of the hepatic macrophage marker F4/80 antibody in vehicle and clodronate liposome administration mice after 70% PH. Scale bars, 20 µM. C) The secretion of IL‐6 was detected from PH, PH+EA, clodronate+PH, and clodronate+PH+EA mice by elisa in the remnant liver at 3 h after 70% PH. D) The mRNA expression of IL‐6 at 3 h after 70% PH in mice from PH, PH+EA, clodronate+PH, and clodronate+PH+EA mice. E) PCNA protein expression in liver tissue at 48 h after 70% PH in four groups by western blot and the relative protein expression analysis. F) IHC staining of Ki67 at 48 h after 70% PH in four groups and the quantification of Ki67 positive cells. Scale bars, 20 µm. Data represent the mean ± SD. ns, *P* > 0.05; *, *P* < 0.05; **, *P* < 0.01; ***, *P* < 0.001. The experiments above were repeated three times.

**Figure 10:**
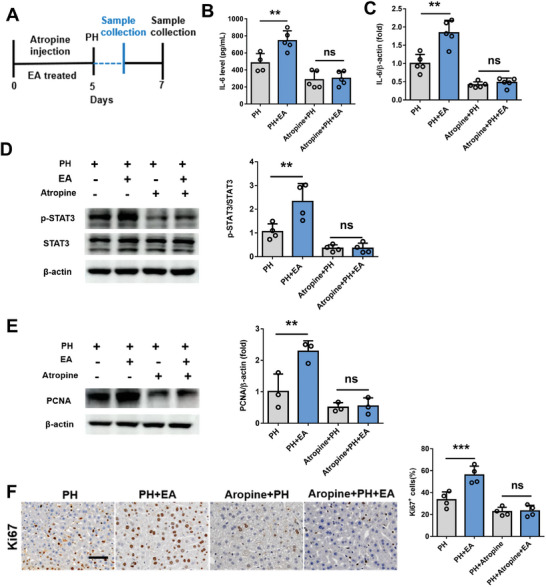
EA promotion of liver regeneration depends on liver cholinergic signaling. A) Schematic of aprotine injection in mice. B) The secretion of IL‐6 was detected from PH, PH+EA, Aprotine+PH, and Aprotine+PH+EA mice by elisa in the remnant liver at 3 h after 70% PH. C) The mRNA expression of IL‐6 at 3 h after 70% PH in mice from PH, PH+EA, Aprotine+PH, and Aprotine+PH+EA mice. D) Western blot assay for phospho‐STAT3, total STAT3, and β−actin protein expression in liver tissue at 48 h after 70% PH in four groups and the relative protein expression analysis. E) PCNA protein expression in liver tissue at 48 h after 70% PH in four groups by western blot and the relative protein expression analysis. F) IHC staining of Ki67 at 48 h after 70% PH in four groups and the quantification of Ki67 positive cells. Scale bars, 20 µM. Data represent the mean ± SD. ns, *P* > 0.05; **, *P* < 0.01; ***, *P* < 0.001. The experiments above were repeated three times.

To further verify the critical role of liver macrophages in the process of EA activating vagus nerve to promote liver regeneration, we examined post PH hepatocyte proliferation using mice in which hepatic macrophages had been depleted by treatment with clodronate liposomes. Clodronate liposome administration significantly decreased the expression of the hepatic macrophage marker F4/80 (**Figure** **9**A,B), indicating successful depletion of hepatic macrophages. We found that clodronate administration attenuated IL‐6 production in both serum and liver in mice at 3 h after 70% PH. It is worth mentioning that clodronate administration completely eliminated the effect of EA on increasing IL‐6 secretion in the early stage of liver regeneration after 70% PH in mice (Figure 9C,D). Correspondingly, according to the protein expression of PCNA and Ki67, the enhancement of EA on the proliferation of residual liver was markedly reduced in clodronate administration mice (Figure 9E,F). Then, we aimed to find the key mechanisms by which vagal signals activate liver macrophages. To examine whether the muscarinic receptor signaling induced by acetylcholine, the main neurotransmitter released by the vagal nerve, is involved, mice were treated with atropine, a muscarinic receptor antagonist, followed by PH. The increases of IL‐6 production, PCNA and Ki67 protein expression were markedly reduced in atropine treatment mice after 70% PH. Interestingly, atropine treatment eliminated the role of EA in promoting IL‐6 secretion from liver macrophages and STAT3 phosphorylation at 3 h after 70% PH (**Figure** **10**A–D), meanwhile, the protein expression levels of Ki67 and PCNA all showed the consistent results above (Figure 10 E,F). Furthermore, we also screened the major acetylcholine receptors expressed in liver macrophages (Figure S3, Supporting Information), and qpcr results showed that EA significantly increased the expression of acetylcholine receptor type m2 (m2AchR). To further illustrate the crucial role of m2AchR, we introduced a specific inhibitor for m2AchR. The results indicated that the enhancement of liver regeneration by EA was abolished after the addition of the m2AchR inhibitor (Figure S7, Supporting Information), suggesting that m2AchR may be the primary acetylcholine subtype involved in vagal nerve function.

## 3 Discussion

The process of liver regeneration is a sophisticated and well‐coordinated physiological phenomenon that is intricately regulated by organisms. Previous research has primarily focused on elucidating the regulatory roles played by factors such as inflammatory mediators, growth factors, metabolic networks, and immune responses in the liver.^[^ ^14^ ^]^ However, the precise impact of external influencing factors, particularly those related to the nervous system, on liver regeneration remains uncertain. The liver is innervated by autonomic and sensory fibers from both the sympathetic and parasympathetic divisions of the nervous system, which play a crucial role in regulating liver function, facilitating liver regeneration, and managing liver diseases.^[^ ^15^ ^]^ The liver performs a multitude of vital functions in maintaining bodily homeostasis, with autonomic and sensory nerve fibers intricately regulating various aspects of hepatic function, repair, and regeneration.^[^ ^16^ ^]^ Although the extent of neural regulation in the process of liver regeneration remains challenging to ascertain, a plethora of studies have demonstrated that liver growth may result from a synergistic interplay between sympathetic and parasympathetic nerves.^[^ ^8^, ^17^ ^]^ In our study, we demonstrated for the first time that EA at ST36 promotes liver regeneration initiation by activating acetylcholinergic neurons in the dorsal motor nucleus of the vagus. This activation leads to the release of acetylcholine from hepatic vagus nerve terminals and synergizes with IL‐6 released from hepatic macrophage cholinergic receptors. These findings not only unveil the pivotal role of the brain‐vagus‐macrophage axis in EA‐induced liver regeneration promotion but also provide a novel therapeutic approach for post‐hepatectomy liver regeneration disorders.

The brain and peripheral organs communicate via hormonal signaling and neural pathways.^[^ ^18^ ^]^ The maintenance of normal whole‐body energy homeostasis necessitates effective communication.^[^ ^19^ ^]^ In addition to the endocrine system, extensive research has been conducted on the role of the sympathetic nervous system in metabolic homeostasis from a neural connectivity perspective; however, our understanding of the parasympathetic nervous system (PNS) remains limited.^[^ ^20^ ^]^ PNS, also known as the vagus nerve, regulates the functioning of peripheral organs such as the liver.^[^ ^21^ ^]^ The dorsal motor nucleus of the vagus is a crucial nuclear mass within the brain. It not only receives sensory input from the vagus nerve, but also integrates and processes this information to regulate visceral movement via parasympathetic efferent fibers.^[^ ^22^ ^]^ It is well known that acetylcholinergic neurons are the major neuron type in the DMV of brain.^[^ ^23^ ^]^ Our study has demonstrated that the liver neurons project onto acetylcholinergic neurons in the DMV region of the brain in mice. Moreover, we have found that chemogenetic inhibition of Ach neurons in the DMV region abolishes the effect of EA on liver regeneration. This suggests that EA promotes liver regeneration by activating acetylcholinergic neurons in the DMV region of the brain. Therefore, our findings indicate that brain DMV acetylcholine neurons play a crucial role in promoting liver regeneration through EA.

The liver is innervated by autonomic and sensory fibers originating from the sympathetic and parasympathetic divisions of the nervous system, which play a crucial role in regulating liver function, promoting liver regeneration, and managing liver diseases.^[^ ^24^ ^]^ The performance of HV has been associated with an increased incidence of postoperative mortality and impediment to liver regeneration.^[^ ^7^, ^25^ ^]^ Repetitively, our findings consistently demonstrate that HV abolishes the hepatoprotective effect of EA, while chemical sympathetic denervation has negligible influence on the hepatic regenerative capacity of EA. Our data further substantiates that EA promotes hepatic regeneration through activation of the vagus nerve rather than the sympathetic nerve. Recently, there have been reports indicating that EA at ST36 can modulate the systemic inflammatory response by activating the vagal‐adrenal pathway.^[^ ^9^, ^26^ ^]^ The findings of our study demonstrated the effective facilitation of liver regeneration in mice undergoing bilateral adrenal excision after 70% PH by EA. Interestingly, our study revealed that EA promotes liver regeneration not through the classical vagus‐adrenal pathway, but rather by directly regulating the vagus nerve and playing a multifunctional role in liver regeneration. Furthermore, our study provided evidence that EA exerts a direct regulatory influence on liver regeneration via the brain‐vagus nerve axis, thereby further substantiating the existence and significance of the brain‐liver axis.

It was reported that vagus nerves in the liver were observed only around the portal region.^[^ ^6a^ ^]^ Therefore, in order to achieve prompt regeneration of residual hepatocytes, it is imperative to establish a distinct mechanism through which neuronal signals can be effectively transmitted to individual liver cells within this vast organ. In this particular context, by leveraging resident macrophages as intermediaries, regenerative signals originating from the vagus nerve can be amplified and disseminated throughout the entire remnant liver via IL‐6 secretion.^[^ ^27^ ^]^ In our study, administration of clodronate completely abolished the effect of EA on increasing IL‐6 secretion and promoting liver regeneration after 70% PH in mice. Our findings highlight the crucial role of liver macrophages in mediating the communication between the vagus nerve and hepatocytes during EA‐induced liver regeneration after 70% PH in mice. Furthermore, IL‐6 serves as a pivotal mediator in facilitating the interaction between liver macrophages and hepatocytes. Our data indicate that EA significantly enhances IL‐6 secretion and STAT3 phosphorylation, a downstream target of its receptor, during the initiation phase of liver regeneration after 70% PH in mice. Conversely, both high vagal tone and depletion of macrophages abolish EA's promotion effect on IL‐6 levels. Interestingly, recent studies have reported divergent inflammatory responses of liver macrophages to cholinergic signals depending on whether muscarinic or nicotinic receptors are activated.^[^ ^27c^ ^]^ Consequently, the response of macrophages to vagus nerve signals may exhibit variability under different physiological or pathological conditions.^[^ ^28^ ^]^ The expression levels of muscarinic and nicotinic receptors on macrophages might also vary in accordance with specific situations;^[^ ^29^ ^]^ however, further research is required to elucidate the molecular mechanisms underlying this contrasting response to cholinergic stimulation. The findings were particularly remarkable as we observed that atropine treatment abolished the role of EA in promoting IL‐6 secretion from liver macrophages and liver regeneration after 70% PH in mice. Moreover, EA significantly enhanced the expression of acetylcholine receptor type m2 (m2AchR), suggesting that m2AchR may be the primary subtype involved in vagus nerve action. To further demonstrate the critical role of m2AchR, we added m2AchR specific inhibitor, and the results showed that the promotion of liver regeneration by EA disappeared after the addition of m2AchR inhibitor(Figure S7, Supporting Information). and the results showed that the promotion of liver regeneration by EA disappeared after the addition of m2AchR inhibitor (Figure S7, Supporting Information). It suggested that m2AchR is the major acetylcholine receptor subtype responsible for EA activation of vagus nerve, leading to the release of IL‐6 from liver macrophages and promoting liver regeneration.

**Figure 11:**
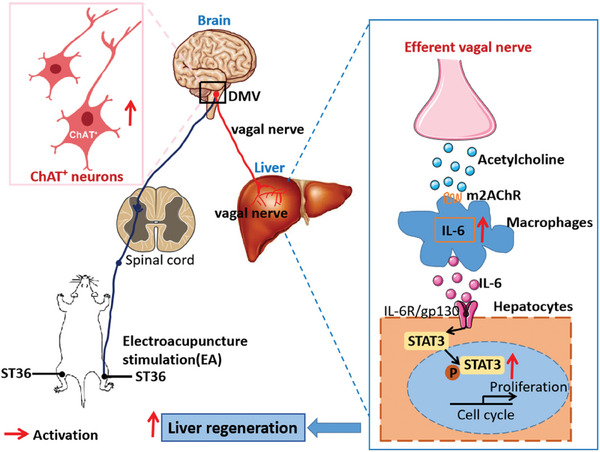
Summary mechanism diagram illustrating EA at ST36 acutely activates cholinergic neurons in the DMV of the brain, resulting in increased release of acetylcholine from hepatic vagus nerve endings and subsequent activation of IL‐6 signaling in liver macrophages.

In conclusion, our study demonstrated that EA at ST36 acutely activates cholinergic neurons in the DMV of the brain, resulting in increased release of acetylcholine from hepatic vagus nerve endings and subsequent activation of IL‐6 signaling in liver macrophages. Ultimately, this promotes hepatocyte proliferation and liver regeneration (**Figure** **11**). Our findings provide further insight into how EA regulates the brain‐liver axis to promote liver regeneration, highlighting acupuncture treatment as an effective intervention for improving liver regeneration disorders in liver surgery.

## 4 Experimental Section

### Mice

Male C57BL/6 mice and ChAT‐Cre mice aged 8–10 weeks were utilized in all experiments. The ChAT‐Cre mice were obtained from The Jackson Laboratory (Bar Harbor, ME). All mice were bred at the Animal Experimental Center of Shanghai University of Traditional Chinese Medicine (SHUTCM, Shanghai, China). Prior to formal experimentation, the mice underwent a one‐week acclimatization period in a specific pathogen‐free room with ad libitum access to standard chow and water. All animal experimental procedures were performed in complying with a protocol approved by the Laboratory Animal Ethics Committee of SHUTCM (approval number: PZSHUTCM2306190002).

### Electroacupuncture Procedure

The surgical procedure was conducted in accordance with the previously described protocol,^[^ ^30^ ^]^ and the electrical stimulation intensity was adjusted to elicit observable muscle contractions. EA was administered at ST36 acupoint using a frequency of 2/100 Hz and an intensity of 0.5 mA for a duration of 15 min per day over a period of 5 consecutive days. Two acupuncture needles measuring 0.16 millimeters in diameter and 7 millimeters in length (0.16·7 mm, Luoya Shanchuan Medical Device Co., Ltd., China) were inserted into the muscle layer at both ST36 acupoints to a depth of 3 mm. ST36 was located on the anterior tibial muscle, approximately one‐sixth distance between the patella and lateral malleolus. Electrical stimulation was delivered using a HANS‐200A acupoint nerve stimulator.

### Surgical Procedures

The experimental procedures were performed exclusively on male mice aged 8–10 weeks. For hepatic vagotomy (HV), an incision was made in the abdominal wall and the stomach was retracted to expose the anterior vagal trunk and hepatic branch. Subsequently, only the hepatic branch was transected using fine forceps. Immediately after HV, a 70% partial hepatectomy (PH) was conducted by securely ligating and resecting the left lateral and median lobes of the liver with 6‐0 silk suture. In sham operations, solely an abdominal incision was made while preserving both liver tissues and hepatic vagus branch intact. Following completion of surgery, meticulous layer‐by‐layer suturing of both abdominal muscles and skin was performed using 6‐0 silk thread. In experiments illustrated in Figure 5A, PH was carried out 7 days after either HV or sham operation for HV. Bilateral adrenalectomy (ADX): The mice were anesthetized and positioned in a prone manner on the dissection table. A posterior skin incision was made along the midline of the back, extending from the last thoracic vertebra to expose the location of the kidney. Adjacent to the kidney, an adrenal gland, approximately the size of a pink mungbean and surrounded by adipose tissue, was identified. The skin incision was then extended to access and remove the contralateral adrenal gland using similar techniques. Animals were administered 0.9% sodium chloride water for 10 days prior to processing for EA treatment and PH model following adrenal resection.

### In Vivo Carbachol Treatment

The carbachol (Nacalai Tesque, Kyoto, Japan) was dissolved in saline to a concentration of 2 mmol l^−1^. Mice were intraperitoneally administered 200 nmol of carbachol (0.1 ml of a 2 mmol l^−1^ solution). Livers were harvested 6 h after the administration of carbachol.

### In Vivo Atropine Treatment

The compound atropine (Sigma) was dissolved in a saline solution containing 10% v/v ethanol to achieve a concentration of 625 mg dl^−1^. Mice were intraperitoneally administered atropine at a dosage of 25 µg g^−1^ body weight, twice daily, starting from 2 days prior to the surgical interventions until euthanasia.

### Immunofluorescence

The mice were intracardially perfused with a 4% (w/v) paraformaldehyde solution and subsequently dehydrated using a 30% sucrose solution for 3 days following anesthesia induction with an overdose of 1% pentobarbital sodium. Antigen retrieval was performed by overnight incubation at 4 °C with the following primary antibodies: ChAT (1:1000, AMAB91130, Merck), TH (1:500, 25859‐1‐AP, Proteintech), c‐Fos (1:1000, 226 008, Synaptic systems). Subsequently, the sections were incubated with fluorescence‐conjugated secondary antibodies (1:1000, Abcam) at room temperature for 2 h. Images were acquired using Olympus vs200 microscope and cell counting was conducted utilizing ImageJ software (version Fiji).

### Immunohistochemistry (IHC) Staining

The liver samples were fixed in 4% formaldehyde for a minimum of 24 h, followed by paraffin embedding and sectioning into slices with a thickness of 4 µm. Dehydration of the liver sections was carried out using xylene, followed by rehydration with graded ethanol. Subsequently, the sections underwent antigen retrieval and were treated with 3% hydrogen peroxide to block endogenous peroxidase activity. To minimize non‐specific binding, the sections were incubated overnight at 4 °C with primary antibodies (Ki67 diluted at 1:500; GB111141; Servicebio) after being exposed to 10% normal goat serum. On the following day, the sections were washed three times with PBS before being incubated for 1 h with secondary antibodies. Finally, images were captured using an Olympus vs200 imaging system.

### Real‐Time Quantitative PCR (RT‐qPCR)

Total RNA extracted from liver tissue was reverse transcribed into cDNA using HiScript II Q RT SuperMix for qPCR (Vazyme Biotech, China). Real‐time PCR was conducted with SYBR GREEN (Vazyme Biotech, China) on a real‐time PCR system. The internal control used was β‐actin. Primer sequences can be found in Table S1 (Supporting Information).

### Western Blotting Analysis

Liver tissues were subjected to a 30 min ice‐cold lysis using CelLytic MT mammalian tissue lysis reagent supplemented with protease and phosphatase inhibitor cocktails from Sigma. Following this, the cell lysate underwent centrifugation at 12,000 rpm for 15 min at 4 °C. The resultant supernatant was employed for Western blotting analysis according to established protocols.^[^ ^31^ ^]^ Primary antibody against PCNA (1:1000, sc‐7907) were provided by Santa Cruz Biotechnology. Antibody against β‐actin (1:5000, 4967) were provided by Cell Signaling Technology.

### Flow Cytometry

The liver immune cells were isolated from the livers of mice undergoing 70% partial hepatectomy and purified through centrifugation at 850 × g using Percoll (GE Healthcare, USA). Residual red blood cells were lysed with Lysing Buffer (BD Biosciences, USA). Subsequently, the liver immune cells were incubated with the following primary antibodies for 30 minu at 4 °C: CD45, F4/80, IL‐6, CD3, B220 (Biolegend). All the antibodies were used equal to 0.125 µg per test for staining. The cell count was determined using the FACS Verse cell sorter (BD Biosciences) and analyzed utilizing FlowJo software (TreeStar).

### Viral Injection in Brain

For viral injection into the brain, mice were deeply anesthetized using isoflurane and subsequently immobilized in a stereotaxic device (RWD) on a pad. A midline scalp incision was performed, followed by drilling a hole in the skull to facilitate the passage of a glass pipette filled with the virus. Subsequently, the virus was injected into the DMV at coordinates mediolateral (ML) 0.25 mm; anterior‐posterior (AP) 7.5 mm; dorsoventral (DV) 3.6 mm from bregma, using an infusion pump (Micro 4, WPI), connected to a glass microelectrode that allowed for controlled delivery of a specific volume of virus at a rate of 35 nl min^−1^. To prevent viral spread after completion of injection, the pipette remained in place for 5 min post‐injection. All viruses were purchased from BrainVTA. For chemogenetic experiments, rAAV‐hSyn‐DIO‐hM4D(Gi)‐EGFP was delivered into the DMV of ChAT‐Cre mice while rAAV‐ DIO‐EGFP virus served as control. For chemogenetic inhibition studies, CNO (2 mg kg^−1^) was administered via intraperitoneal injection daily for two consecutive days.

### In Vivo Electrophysiological Recording

After a period of adaptive feeding lasting 3 days, a total of 16‐channel electrodes were surgically implanted into the DMV of the mouse brain. Simultaneous recordings from multiple neurons were conducted using a custom‐made spiral‐driven microdriver. Prior to signal recording, the mice underwent a recovery period of at least three days and acclimated to the cables attached to their head‐mounted electrodes. All neural recordings were performed concurrently utilizing the OmniPlex system for data acquisition (Plexon).

### Liquid Chromatography‐Tandem Mass Spectrometry (LC‐MS)‐Based Quantification of Neurotransmitters in Liver of Mice

The liver tissue of mice was homogenized in a methanol solution, followed by centrifugation at 4 °C and 20000 × g for 15 min. The resulting supernatant was collected, and subsequently, 1 µL of the bisected solution was injected into the LC‐MS system. The method for detecting neurotransmitters as described previously.^[^ ^32^ ^]^

### Statistical Analysis

All the details about sample size, data presentation, statistical analysis, and significant differences are provided in respective figure legends. Experiments were conducted three times at least and all data were presented as mean ± SD. Data analyses were performed through GraphPad Prism 6. The difference between two groups was examined using un‐paired student test. All the comparisons among multiple‐groups were analyzed by one‐way ANOVA test. Exact P values of statistics analysis were directly shown on the respective Figures, P‐values < 0.05 were regarded as statistically significant.

## Conflict of Interest

The authors declare no conflict of interest.

## Author Contributions

L.Y., Y.Z., and Z.H. contributed equally to this work. L.Y. designed the project and wrote the manuscript. L.Y., Y.Z., and Z.H. performed experiments with the help from W.L., J.L. and W.H. Y.S., F.W., and X.S. interpreted the data. J.S., H.W., and X.K. revised the completed manuscript. The final manuscript was read and approved by all the authors.

## Supporting information

Supporting Information

## Data Availability

The data that support the findings of this study are available from the corresponding author upon reasonable request.
