# Supplementary material for: Electroacupuncture Promotes Liver Regeneration by Activating DMV Acetylcholinergic Neurons‐Vagus‐Macrophage Axis in 70% Partial Hepatectomy of Mice
Source: Adv Sci (Weinh). 2024 Jun 25;11(32):2402856. doi: 10.1002/advs.202402856 (PMC11348175; doi:10.1002/advs.202402856)
Supplement: Supplementary file 1 — Supporting Information [file ADVS-11-2402856-s001.doc]

**Supporting Information**

Electroacupuncture Promotes Liver Regeneration by Activating DMV Acetylcholinergic Neurons-Vagus-Macrophage Axis in 70% Partial Hepatectomy of Mice

Liu Yang^1^**^#^**, Yanyu Zhou^1^**^#^**, Zhaoshuai Huang^4^**^#^**, Wenxuan Li^1^, Jiacheng Lin^1^, Weifan Huang^1^, Yali Sang^1^, Fang Wang^1^, Xuehua Sun, Jiangang Song^2^*, Hailong Wu^3^*, Xiaoni Kong^1^*

1 Central Laboratory, Shuguang Hospital Affiliated to Shanghai University of Traditional Chinese Medicine, Shanghai, China.

2 Department of anaesthesiology, Shuguang Hospital Affiliated to Shanghai University of Traditional Chinese Medicine, Shanghai, China.

3 Shanghai Key Laboratory of Molecular Imaging, Collaborative Innovation Center for Biomedicines, Shanghai University of Medicine and Health Sciences, Shanghai, China.

4. Abdominal Transplantation Center, General Surgery, Ruijin Hospital, School of Medicine, Shanghai Jiao Tong University, Shanghai, China.

Authorship note: **^#^** These authors contributed equally to this study.

*Corresponding authors:

Xiaoni Kong, Ph.D., Central Laboratory, Shuguang Hospital Affiliated to Shanghai University of Traditional Chinese Medicine, 528 Zhangheng Road, Shanghai, China, 201203, Email: xiaoni-kong@126.com; or Hailong Wu, PhD, [wuhl@sumhs.edu.cn](mailto:wuhl@sumhs.edu.cn); or Jiangang Song, MD, [songjg1993@shutcm.edu.cn](mailto:songjg1993@shutcm.edu.cn)

**Experimental Section**

*In Vivo Levetiracetam Treatment:* The compound levetiracetam (Felix, cat: FB07982) was dissolved in PBS. Mice were intraperitoneally administered levetiracetam at a dosage of 50mg/kg body weight, once daily, starting from 3 days prior to the surgical interventions until euthanasia.

*Bilateral Adrenalectomy (ADX):* The mice were anesthetized and positioned in a prone manner on the dissection table. A posterior skin incision was made along the midline of the back, extending from the last thoracic vertebra to expose the location of the kidney. Adjacent to the kidney, an adrenal gland, approximately the size of a pink mungbean and surrounded by adipose tissue, was identified. The skin incision was then extended to access and remove the contralateral adrenal gland using similar techniques. Animals were administered 0.9% sodium chloride water for 10 days prior to processing for EA treatment and PH model following adrenal resection.

**
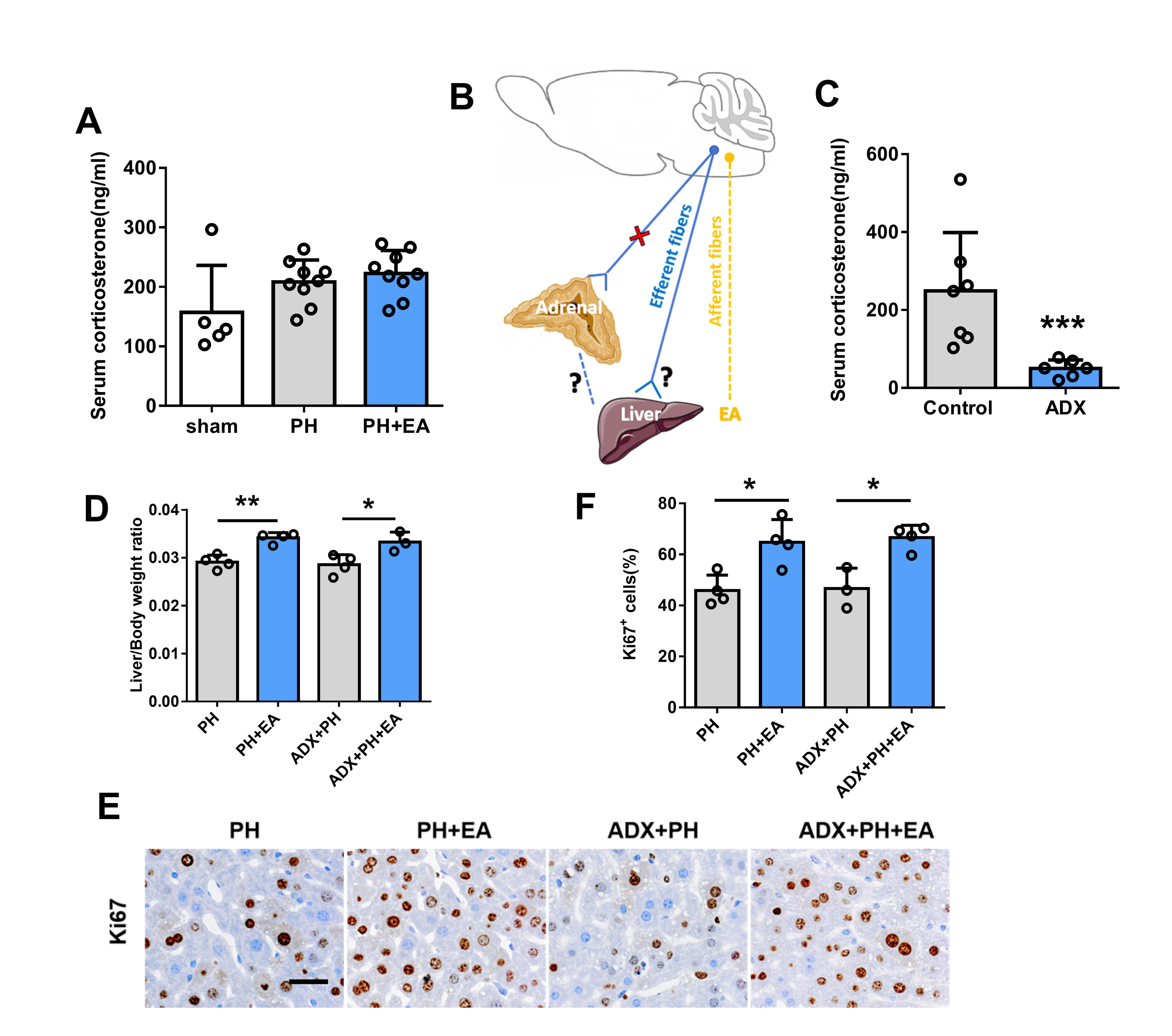
**

**Figure S1.** EA directly regulates liver regeneration through the vagus nerve rather than indirectly through the vagal-adrenal axis. A) Mass spectrometry detection of the content of serum corticosterone in sham, PH, and PH+EA groups after 70% PH. B) Schematic of bilateral adrenalectomy (ADX) in mice. C) Mass spectrometry detection of the content of serum corticosterone in control and ADX mice. D) liver/body weight ratio of mice in PH, PH+EA, ADX+PH, ADX+PH+EA groups at 48h after 70% PH. E and F) IHC staining of Ki67 48h after 70% PH in four groups and the quantification of Ki67 positive cells. Scale bars, 20 μM. Data represent the mean ± SD. *, P < 0.05; **, P < 0.01; ***, P < 0.001. The experiments above were repeated three times.


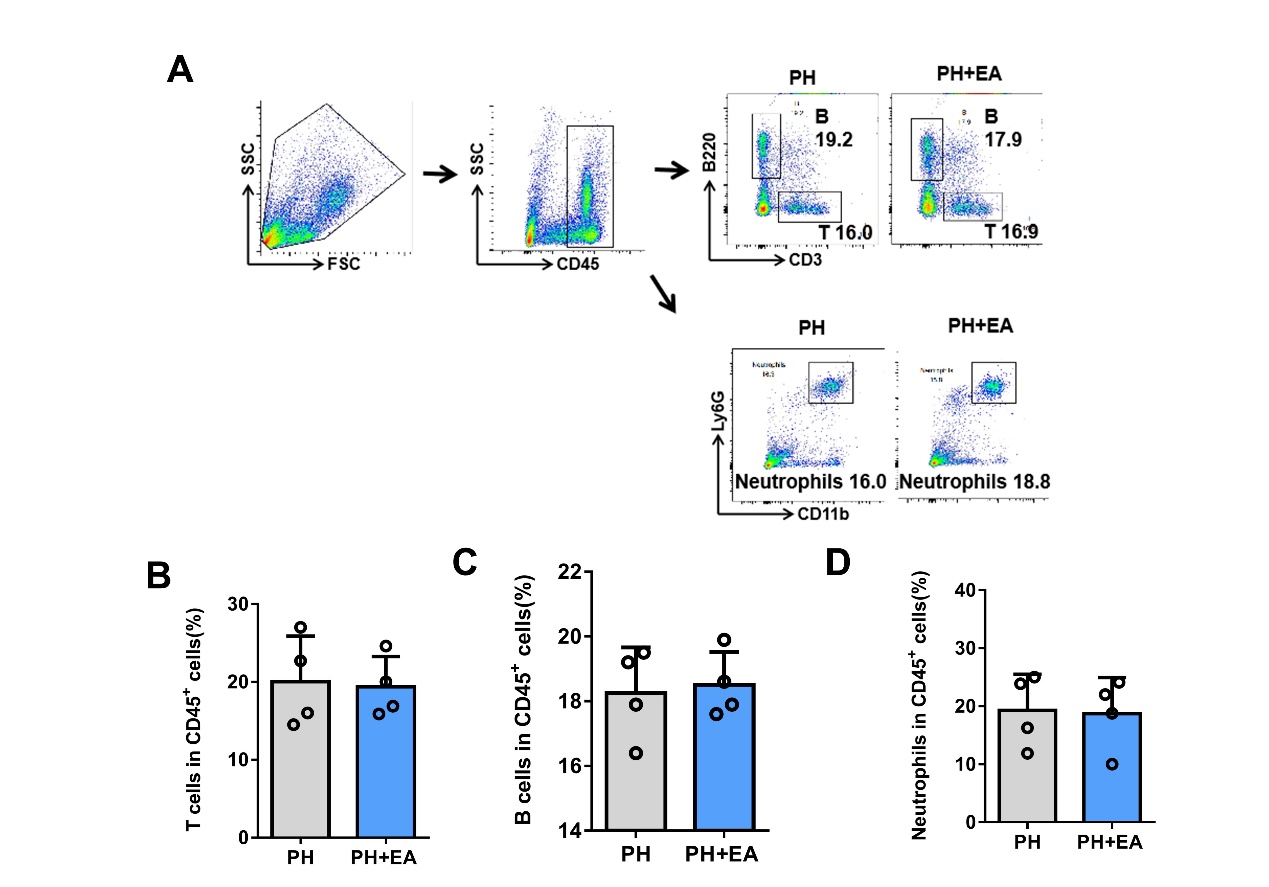


**Figure S2.** EA had no effect on T, B and neutrophils ratio in the early stage of liver regeneration after 70% PH. A) Representative FACS plots of neutrophils, B and T cells from PH and PH+EA mice at 3 h after PH and (B, C and D) quantification of the proportion of these immune cell subtypes did not differ significantly.


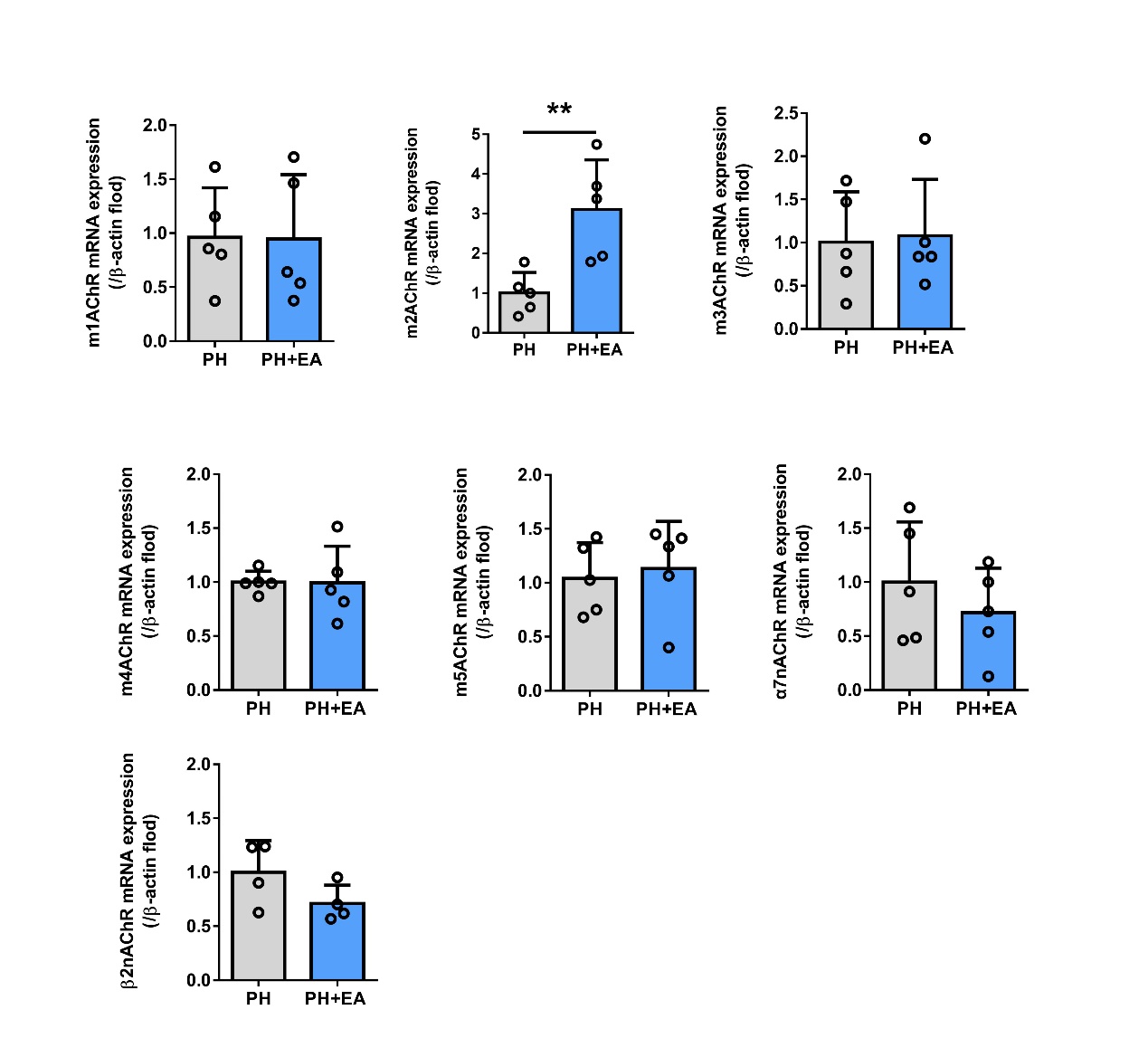


**Figure S3.** Differential genes of EA treated acetylcholine receptor were screened in liver macrophages by qpcr assay. mRNA expressions of AchRs in liver tissue 3h after PH in PH and PH+EA mice, including the genes of m1AchR, m2AchR, m3AchR, m4AchR, m5AchR, α7nAchR and β2nAchR. Data represent the mean ± SD. **, P < 0.01. The experiments above were repeated three times.


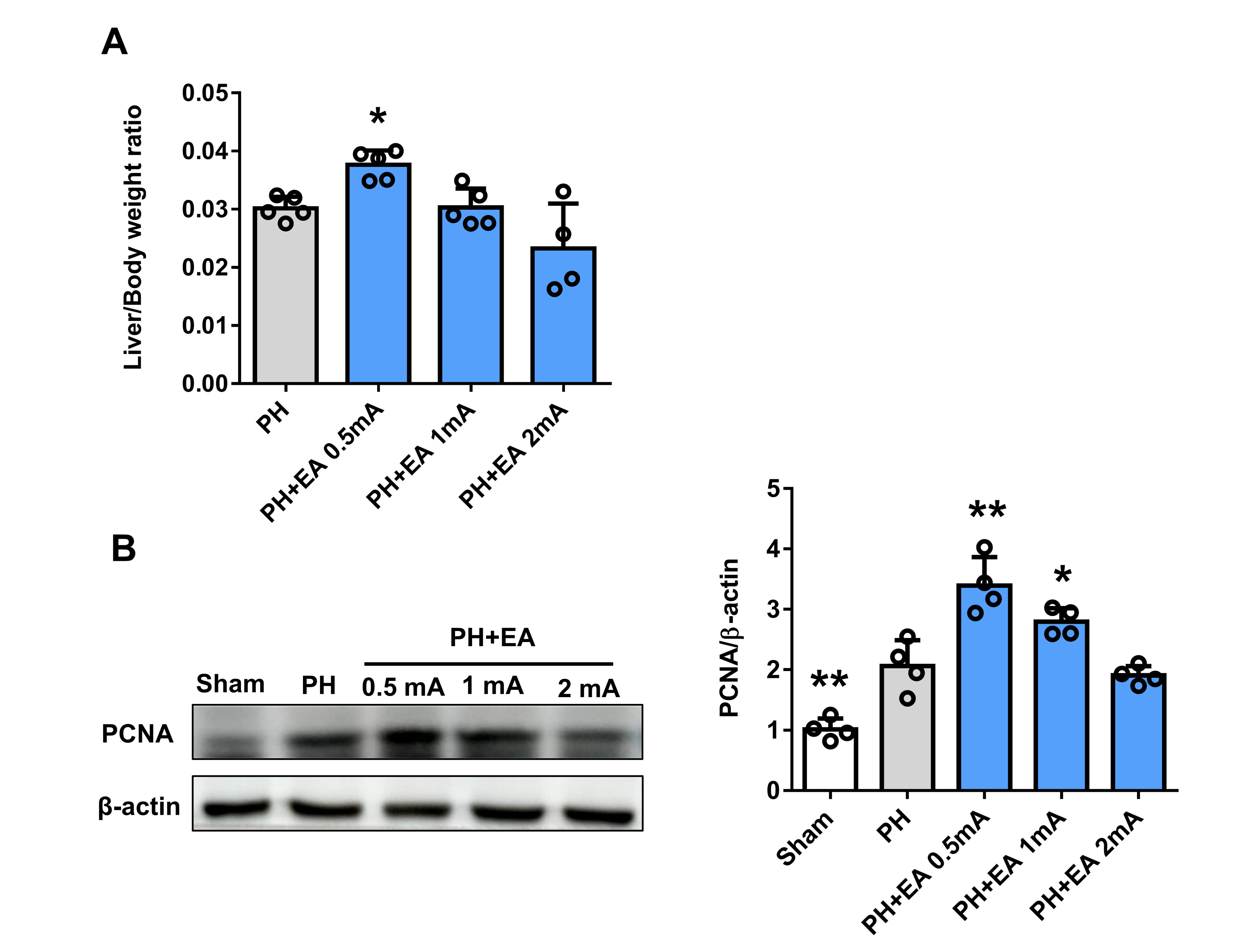


**Figure S4.** The effects of different EA intensities on liver regeneration at 48 hours after 70% PH.

1. Liver/body weight ratio of mice in different EA intensities after 48h 70% PH. B) PCNA protein expression in liver tissue at 48h after 70% PH in different EA intensities by western blot and the relative protein expression analysis. Data represent the mean ± SD. *, P < 0.05; **, P < 0.01. The experiments above were repeated three times.


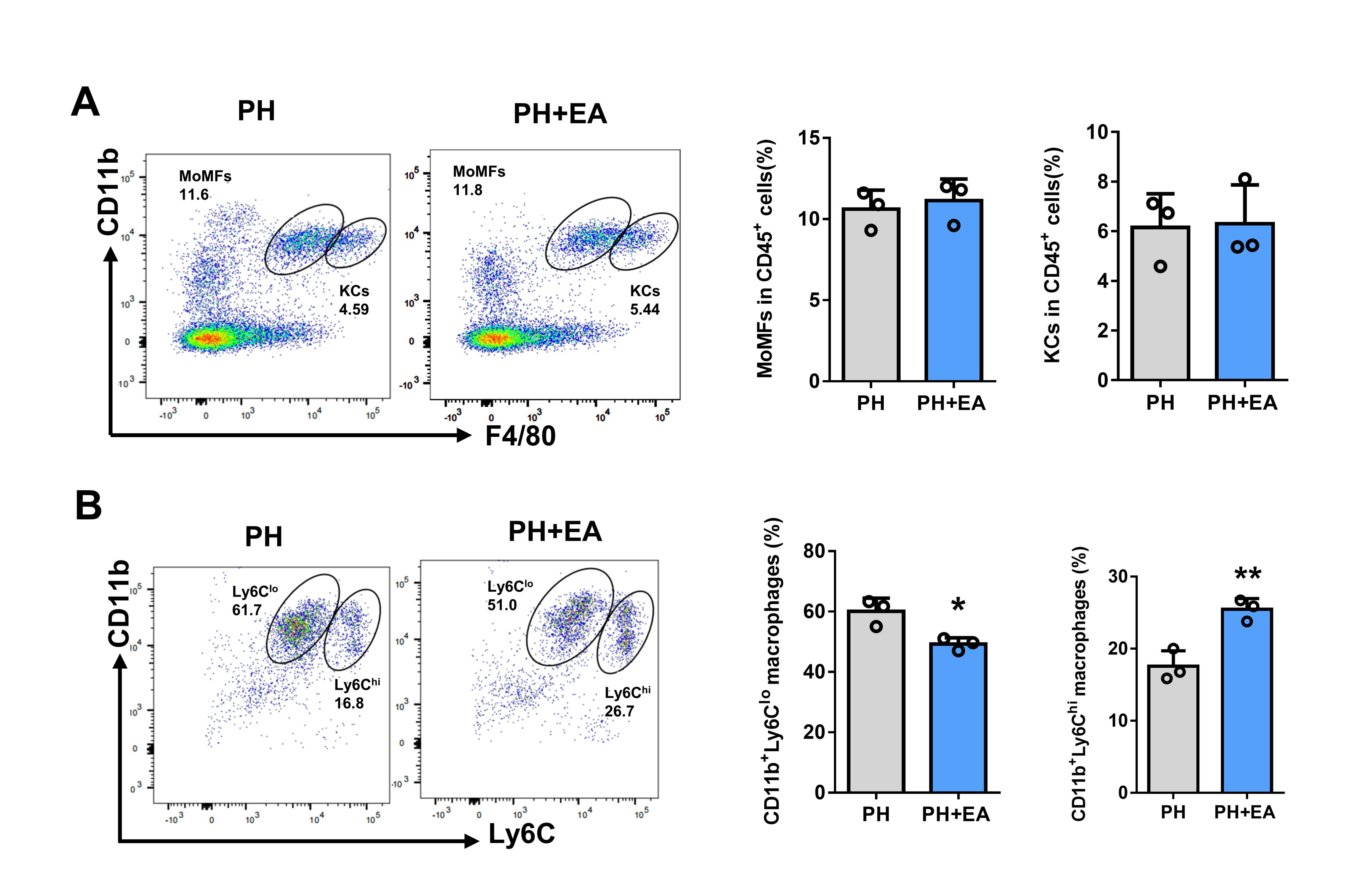


**Figure S5.** Effects of EA on types (KCs and infiltrated macrophages) and subtypes ( (M1/M2)) of liver macrophages. A) Representative FACS plots and the quantification of MoMFs and KCs in liver at 3 h after 70% PH. B) Representative FACS plots and the quantification of Ly6C^lo^ or Ly6C^hi^ hepatic macrophages 3 h after 70% PH. Data represent the mean ± SD. *, P < 0.05; **, P < 0.01. The experiments above were repeated three times.


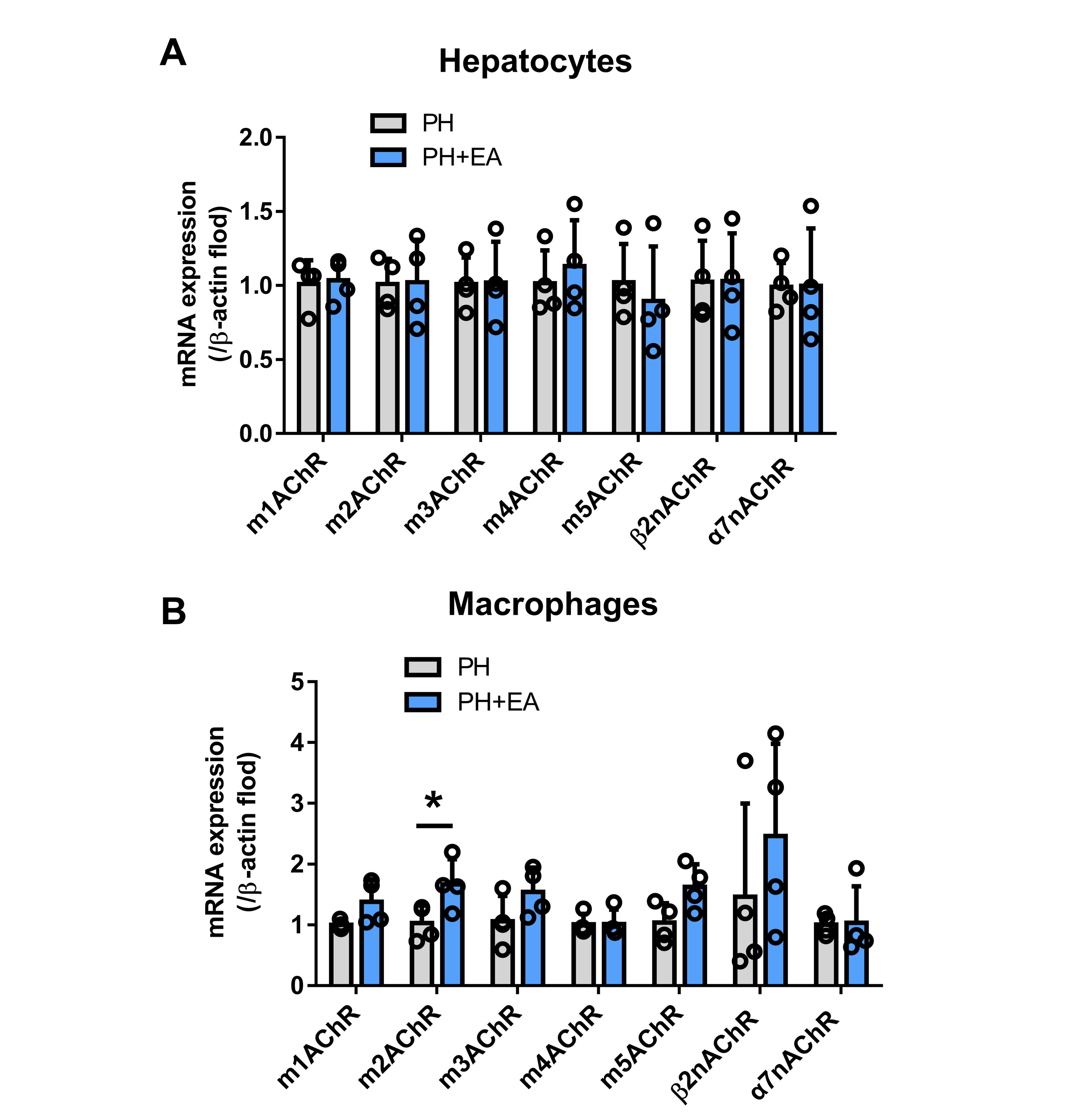


**Figure S6.** Differential genes of EA treated acetylcholine receptor were screened in macrophages or hepatocytes in the liver separately by qpcr assay. A, B) mRNA expressions of AchRs in hepatocytes and macrophages 3h after PH in PH and PH+EA mice separately, including the genes of m1AchR, m2AchR, m3AchR, m4AchR, m5AchR, α7nAchR and β2nAchR. Data represent the mean ± SD. *, P < 0.05. The experiments above were repeated three times.


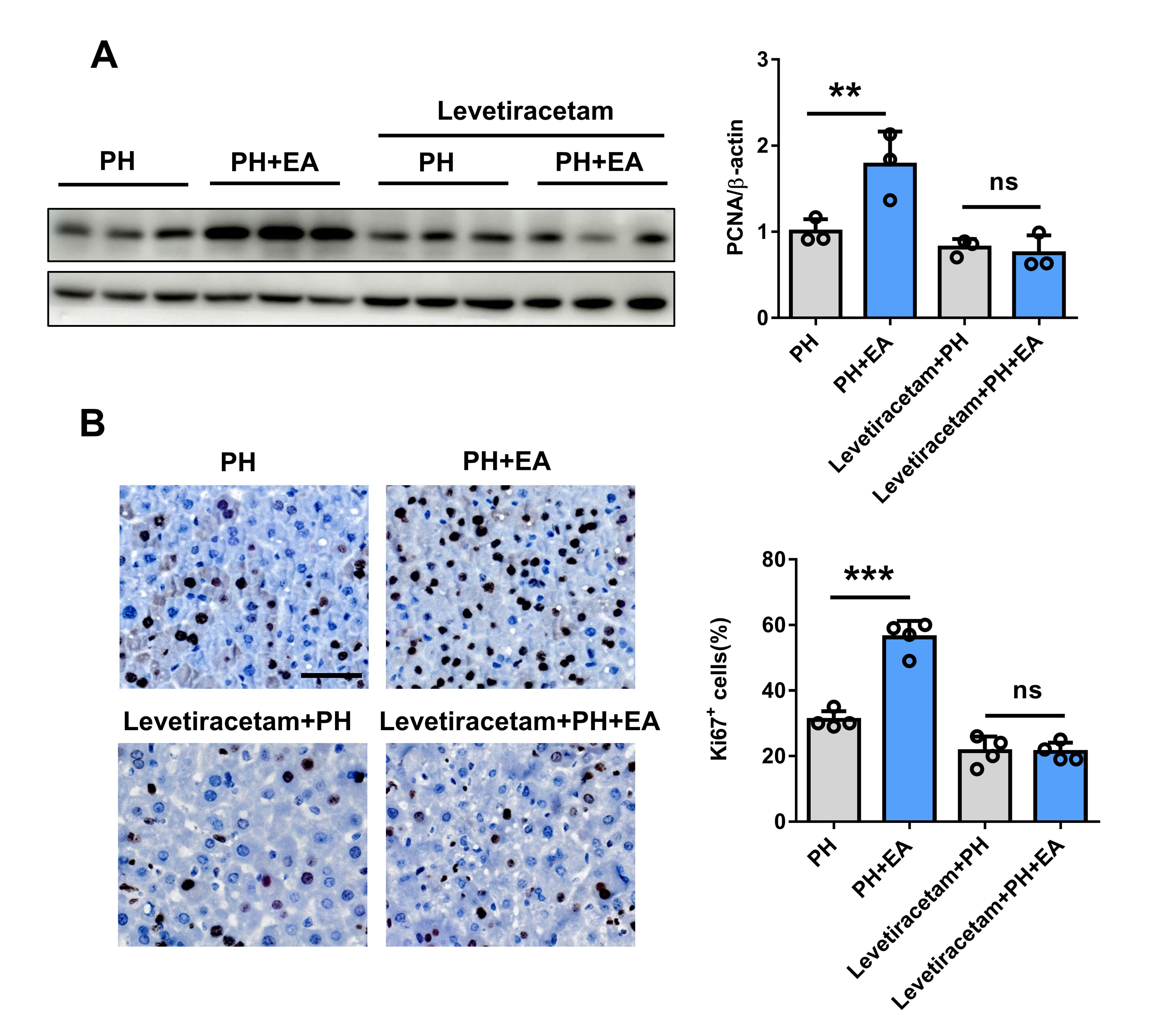


**Figure S7.** The m2AchR specific inhibitor administration eliminated the role of EA in promoting liver regeneration after 70% PH. A) PCNA protein expression in liver tissue 48h after 70% PH in four groups and the relative protein expression analysis. B) IHC staining of Ki67 48h after 70% PH in four groups and the quantification of Ki67 positive cells. Scale bars, 20 μM. Data represent the mean ± SD. ns, P > 0.05; **, P < 0.01***; P < 0.001. The experiments above were repeated three times.


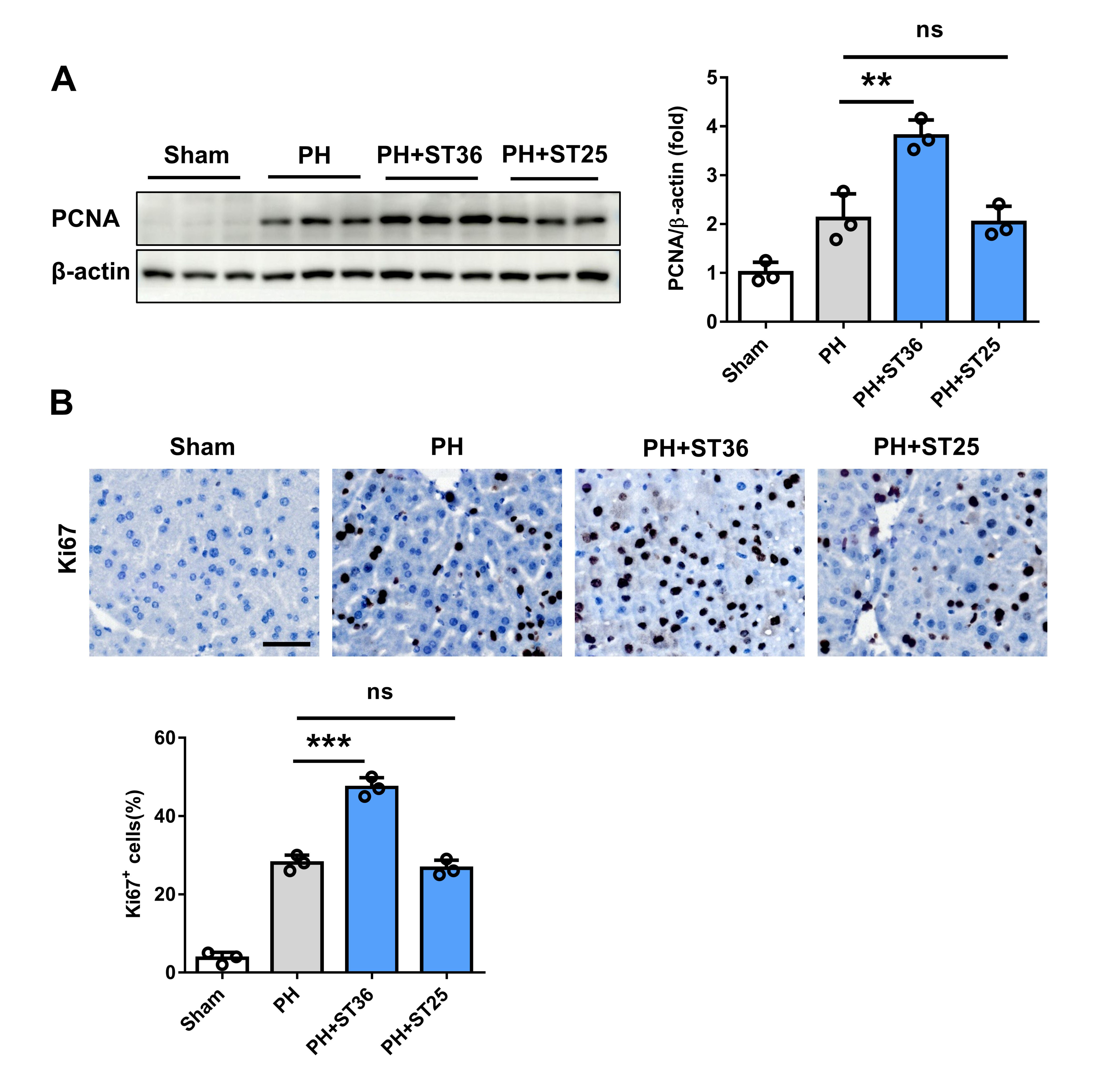


**Figure S8.** EA at ST36 was more effective in promoting liver regeneration than ST25. A) PCNA protein expression in liver tissue 48h after 70% PH in sham, PH, PH+ST36 and PH+ST25 groups. B) IHC staining of Ki67 48h after 70% PH in four groups and the quantification of Ki67 positive cells. Scale bars, 20 μM. Data represent the mean ± SD. ns, P > 0.05; **, P < 0.01***; P < 0.001. The experiments above were repeated three times.

**Table S**1. Sequences of primers for real-time PCR analysis.

| Gene | Forward primer | Reverse primer |
| --- | --- | --- |
| β-actin  Chat  IL-6  TNF-α  PCNA  CyclinA2  CyclinB1  CyclinD1  CyclinE1  m1AchR  m2AchR  m3AchR  m4AchR  m5AchR  α7nAchR  β2nAchR | GTGACGTTGACATCCGTAAAGA  AGG GCA GCC TCT CTG TAT GA  TCACAGAAGGAGTGGCTAAGGACC  CCCTCACTAAGATCT  TTTGAGGCACGCCTGATCC  GCCTTCACCATTCATGTGGAT  AAGGTGCCTGTGTGTGAACC  GCGTACCCTGACACCAATCTC  GTGGCTCCGACCTTTCAGTC  AGTCCCAACATCACCGTCTTG  TGGTTTGGCTATTACCAGTCCT  CCTCGCCTTTGTTTCCCAAC  ATGGCGAACTTCACACCTGTC  TCAACGGCACCCCAGTAAATC  CACATTCCACACCAACGTCTT  AGGGGTTTTGGGTACTGACAC | GCCGGACTCATCGTACTCC  GGC TCA GGC ATG ATA CTG CT  ACGCACTAGGTTTGCCGAGTAGAT  GCTACGTGGGCTACA  GGAGACGTGAGACGAGTCCAT  TTGCTGCGGGTAAAGAGACAG  GTCAGCCCCATCATCTGCG  CTCCTCTTCGCACTTCTGCTC  CACAGTCTTGTCAATCTTGGCA  CAGGTTGCCTGTCACTGTAGC  CTGAAGGTGGCGGTTGACTT  TTGAGGAGAAATTCCCAGAGGT  CTGTCGCAATGAACACCATCT  GGATGTAGGTCGTGTAGAGGTTC  AAAAGGGAACCAGCGTACATC  AGCTTGTTATAGCGGGAAGGA |
